# Can open science follow open access?

**DOI:** 10.1016/j.jbc.2021.101473

**Published:** 2021-12-16

**Authors:** Alex Toker

As I started mulling over contributing my first editorial since becoming the editor-in-chief for JBC on October 1st 2021, I kept coming back to the remarkable winds of change that have been blowing at the journal and the ASBMB during the past year. I am a firm believer that change is good—it can be a driving force for forward progress—and in this regard, JBC has truly excelled. Under the brilliant guidance of the former editor-in-chief, Prof. Lila Gierasch and the ASBMB leadership, JBC has gone fully gold open access as of January 1, 2021 (1). This means that in addition to upon acceptance and publication as a paper-in-press, which JBC has always provided to all readers regardless of subscription, all fully redacted articles are available to everyone around the globe, for free. You do not need a personal or library subscription to view all published JBC content, you simply need an internet connection. I have become a supporter of open access publishing, and it is one of several reasons why I accepted the JBC EiC position. This also reminds me that not only myself, but indeed all of us in the JBC community owe a tremendous debt of gratitude to Lila Gierasch for steering the journal through this momentous transition and also to Fred Guengerich who served as interim EiC until I took over.

The move to gold open access has had inevitable consequences for the journal and, importantly, for the ASBMB. All of us believe that 2021 was the right time to make the move to open access, with the ever-changing face of the publication industry, the implementation of Plan S (https://sparcopen.org/news/2018/coalition-european-funders-announces-plan-s/), and the requirement by many funding agencies that their sponsored research must be published in an open access-compliant journal. As a consequence, the relationship with Elsevier as the commercial partner was initiated. It is important to recognize that the full editorial control of all articles remains with the editors and staff at JBC. JBC remains, at its core, a journal *‘for scientists, run by scientists’*. JBC and ASBMB also continue to perform data integrity analysis on all accepted articles ahead of formal acceptance, one of the few journals to do so. For these reasons, JBC remains a society-owned and a society-run journal, whose primary goal is to disseminate science and foster a community of scientists working in all areas of cell and molecular biology and biochemistry.

Why publish in a society-based journal? Historically, this was a straightforward answer. Academic, or ‘learned’ societies serve their constituent members through their many activities, not only publishing papers but also hosting conferences, science advocacy, public affairs, and other activities designed to foster communities. The reality is that this has not changed in the 21st century. What has changed is the landscape of journals, many designed in the for-profit model. There is nothing necessarily wrong with for-profit publishing, and indeed many commercial publishers do pump money back into science in a variety of useful ways. Moreover, it costs money to handle and publish scientific results. Indeed, the primary motivation for the gold open access model is that the cost of funding and doing science and then publishing the same science should not be the burden of the taxpayer, at least in the case of the NIH and other government agencies. Why pay to read a scientific study, when you already paid to fund that study? Of course, it is a much more complex proposition, and this I believe is where academic societies can fundamentally help their constituent members, because they are not-for-profit organizations. The article processing charge (APC) for JBC is $2000 if you are an ASBMB member or $2500 if you are not. It is hugely important to recognize that (1) this APC is one of the lowest in the journals’ marketplace and (2) the proceeds garnered from the APC for every single JBC paper goes back to the ASBMB for its many scientific activities. So, not only is it much more cost-effective to publish in JBC; in addition, a major proportion of those dollars go straight back to promoting science. The move to open access and partnership with Elsevier has led to many changes, mostly behind the scenes, but the core values of JBC, instilled over 100 years ago, have not changed. To hammer the point home, JBC is still a society-run and society-based journal, *for* scientists and *run* by scientists.

What does the immediate future hold in store? The implementation of open access raises an equally important aspect of science publishing in 2021 and beyond – *open science*. The basic premise of open science is that not only should articles be freely accessible to all, but that the primary data contained within them should also be readily available. JBC certainly adheres to this principle, in so much that JBC policy states that all primary data should be available upon request from the authors. The open science movement has gained significant traction over the last decade, and the basic tenets are that articles and data should be published in the fully open access model. Similarly, all primary data, both ‘negative’ and ‘positive’, should be deposited in publicly-accessible repositories, free to all. This is not a particularly heretical concept, after all at JBC and in many other journals, large data sets such as proteomics, RNAseq, functional genomics, and structural data must all be deposited into one of many public repositories as a condition of article acceptance. Why should this not apply to all primary data?

There are numerous ways to define the Open Science movement. One of the most illustrative is the sociologist Robert Merton’s norms from 1942, defining the ethos of science: *Communality*, *Universalism*, *Disinterestedness*, and *Organized Skepticism* (2). *Communality* represents the open and transparent sharing of knowledge, the basic tenet of Open Science. *Universalism* encompasses objective assessments where decisions are driven by data. *Disinterestedness* speaks to motivation, a scientist should be motivated by knowledge and discovery, which to many of us seems self-evident but in practice is often distorted because of external forces. Finally *organized skepticism*, where one limits bias and considers all new evidence, even against one’s prior work. After all, the basic premise of the experimental method has always been one in which scientists define a hypothesis and set out experimental approaches to disprove that hypothesis. All of these idealized norms converge, in collaboration with the scientific method, to ensure a high quality of science. I will be looking forward to discussing with the JBC community the ideals and implementation of Open Science.
